# Moral Insanity in Relation to Criminal Acts

**Published:** 1861-12

**Authors:** I. Parigot

**Affiliations:** Late Commisioner in Lunacy and Chief Physician of the Colony of Gheel, Belgium; Honorary Professor of the University of Brussels; Member of Several Academies and Learned Societies


### SELECTED.

### MORAL INSANITY IN RELATION TO CRIMINAL ACTS.

By I. PARIGOT, M. D.,
*Late Commisioner in Lunacy and Chief Physician of the Colony of Gheel, Belgium; Honorary Professor of the University of Brussels; Member of Several Academies and Learned Societies.*

It is easy to say, *That man is crazy;* but it is not so to prove it. How can a simple affirmation or a medical certificate, without proofs that a man is insane, deprive him of his liberty?

There is no doubt, at least in Europe, and perhaps here, that if mental diseases had been accurately described—if physicians had not contented themselves, and magistrates been satisfied with a single line assuming, *ex virtute officii*, that a man was to be shut up as insane, many errors, injustices, and miseries whould have been spared!

It is true an authentic demonstration is not always easy—who is infallible? At all events, when a physician gives his opinion in a court, relating to the life, honor, and property of individuals, his office is of the highest character. The importance of his function must elevate his soul and mind above all private interest.

As we have said, it is difficult to say when spiritual causes have sufficiently acted upon our body to elicit insanity. Where is the line or boundary of sanity and madness in a corrupted conscience? Here again our only criterion is medical observation, with this difference, that we ought to be very cautious, and always avoid appearing to be under the influence of some secret motive. It is about the same for everybody concerning the moral cause of insanity. There is also a line we ought not to pass. Every man should take care of his weak side. Education should have, for one of these objects, to fortify our wills, in order to make up for gifts that we may want. Not only is this true, but, as the Scriptures say, the

*spirit is the same, but gifts are different.* Let us suppose that a man of ordinary ability would, from the plough, begin and assiduously prosecute high and profound studies. (I have often seen such cases in rural schoolmasters attempting to study divinity.) Now that man will soon feel some premonitory symptoms of an over-exertion and fatigue of his intellectual powers. If he continues his foolish task, he will perhaps very soon say that some light from heaven has descended upon him, and turn a furious maniac.

The so-bitterly criticised theory of a most distinguished alienist, Dr. Moreau de Tours, who asserts that madness is often the result of an over-activity of the brain, is justified in this and many other causes. When that author affirms that if vital force accumulates in one organ, one of its results must be either a great energy of function or a lesion of that organ, he says something very true. It is in this sense that Dr. Moreau finds some affinity between genius and madness. His antagonist, M. Flourens, though a man of immense talent, is, I fear, a bad judge of the value of this theory, since the learned Perpetual Secretary of the Institute of France, amongst a great number of pamphlets on all sorts of subjects, has written a small compilation entitled *Essai physiologique sur la Folie*, which shows that he never gave much attention to that special subject of medicine.

As insanity cannot exist without a form of exterior disease, so bodily health cannot accompany and is incompatible with a mental infirmity. Symptoms of diastrephia are numerous, and moral insanity or diastrephia presents several phases or stadia during which the following symptoms may be observed:

Generally patients are different in manners, ideas, feelings, conduct, and even language, from what they were before. No delirium or delusion is sometimes perceptible. In some cases bad habits, customs, or indulgences, are more frequent and become notorious. After a certain time elapses since the premonitory symptoms, patients become incapable of serious occupations. Their will is impaired, their power of control lost, and their instincts let loose; there appear irresistible or unaccountable eccentricities and whims. It is in this period that sometimes delusions or a mono-delirium appears, but it is rare.

During that phase it has been noticed that some patients take pleasure in telling stories, intriguing and deceiving people; otherwise they are not loquacious; but then the moral infirmity augments. There exist often hardness of heart, selfishness, and bad dispositions. From that state they become profoundly melancholic.

If the character of the disease still augments, immortality accompanies the propensity of doing harm or committing some atrocious crime. The patient is dull, and appears indifferent, though secretly he has a delight of preparing some evil action. When crimes are committed, their execution is particularly remarkable. When the patient is detected, or when he offers himself, he admits readily the facts, however scandalous or horrible they may be. Generally there is a complete absence of grief or remorse.

In all these cases, letters, memoirs, designs, etc., are of the greatest value to ascertain the mental condition of the writer. Their general feature shows cunning, complaints, and false statements.

Diastrephia may have a milder course, and present a remittent series of fits. Some take place several times in the day, or in the week. I have seen a patient who remained sometimes one whole year free from attacks. During their lucid intervals they offer no signs of insanity, except some disturbances in their general health; but in the attack patients say they suffer a great deal in the head.

Some patients, and especially dipsomaniacs, employ much art in preventing people from remonstrating. For instance, they will feel offended—being gentlemen or gentlewomen—to be told that they were found dead drunk in some sewer of a street, etc. Their infirmity makes them powerless against a subsequent impulse, and they fall to the lowest scale of degradation. Some patients, after a fit, regret what they have done, and sometimes beg to be taken care of for the future.

No medical man denies to-day the solidarity of *innervation*, *sanguification*, and *nutrition*, and there is certainly an intimate relation of these functions with those of the brain. For instance, any long process of enervation, produced by solitary habits, will soon operate a change in the mental organism, just as any deviation of nutrition will produce a morbid diathesis.

Who would maintain that a mental lesion can exist exclusively of any morbid reaction of the body? We have never met with such an exception. If it was so, diastrephic cases might remain the subject of interminable discussion between lawyers, philosophers, and physicians. Our conviction on this subject may perhaps make us appear to overrate the value of physical symptoms; but we must declare that it is their coexistence with mental aberration that gives them the advantages upon which we insist.

Now what we say of diastrephia is applicable to cases of simulation of insanity. Let the part of a feigner be played as well as possible, emotions would be difficult to imitate; but somatical symptoms would be still more difficult or impossible to imitate. Supposing even that a simulator could impose upon a skillful observer, the result of his game would very probably be *real* insanity—a curious and forgotten form of insanity to which I shall afterwards refer. For the present I wish to state that it is a direct proof of the power of ideas on our bodily structure.

Among the physical signs which I am going to mention, one of the most important is the existence of a morbid action on the brain. Pain is reported to exist in different parts of the head, and other bodily symptoms depend often upon a reaction of the nervous centres. They present various features, the *ensemble* of which strikes at first sight the practical observer.

According to the excellent observation of Dr. Billod, in the tenth volume of the *Annales medico-physiologiques*, there exists a curious interruption of volition in the muscular system. In spite of the patient his limbs cannot obey his purposes. It is just the opposite of convulsive muscular action which takes place in various diseases. The cerebro-spinal system is disordered, so are the functions of the sympathetic. Irregular innervation of arteries and veins produces latent disorders in various parts, of which patients rarely complain. There is an irregular visceral and capillary circulation which affects nutrition and several of its functions. Patients are emaciated, they feel a general heaviness over the whole body. Their complexion is sallow, their skin harsh, and its emanation has a peculiar smell. Very often they are feverish, the heat of the skin is increased, the pulse frequent, the tongue furred, and the bowels almost permanently confined. Their rest is troubled during the night; they want to lie down often during the day. In cases connected with melancholy there is a deficient sensibility of the peripherical ramification of nerves sometimes producing anæsthesia. Generally an expression of pain is apparent. The physiognomy is dull, and suggestive of indifference and selfishness. The face is pallid; the stare is not vacant but uncertain. Sometimes the eyes wander about, or there exists a tremulous movement of both eyes when the patient fixes them on any one. Squinting has been remarked, but it is rare. Sometimes the pupils are irregularly contracted, which is a bad sign. When there is irritation of the brain the pupils are contracted. They are dilated in the congestive state, without irritation of that organ. The non-contraction of pupils indicates a loss of sensibility of the whole nervous system. Generally there is a peculiar look in diastrephia, indicating shrewdness and a disposition to do mischief. These signs are more apparent when fits are going to take place.

In this disease we have seldom seen a case without dyspepsia; there are gastralgic pains and a voracious appetite. Palpitation of the heart very often accompanies that disposition, and augments the sufferings of the patient. Complications may arise: thus uterine affections are the source of moral disturbances. Some arise from essential and local diseases, as gout and rheumatism.

Patients of a nervopathic disposition are often perfect hypochondriacs. Some, in spite of a suicidal propensity, are constantly occupied with their health and comfort. They ask continually for medicines, and are afraid of doing something wrong concerning some trifle, but, by a sudden impulse, they will commit suicide. When interrogated or asked why they committed certain deeds, some can give no reason or account for it; it was *an impulse*, *a desire*, etc. Some are very cunning in avoiding explanations.

We have seen a lunatic who wanted to be under the guard of somebody, even of a child, lest he should do some harm. That man, who had resided several years in the village of Gheel, in Belgium, was boarded in the family of a small farmer, and had never committed the slightest offence although under the influence of distrephia. I often visited him; he was sometimes alone, or under the guard of children—the parents being at work in the adjoining fields. Some people of his birthplace having told the burgomaster of his village that the patient was cured, since no insanity could be found in his *talking*, a medical report was required, and, notwithstanding its conclusions, the administrative authority set him at liberty. He went home; but the man, let loose to his propensity to murder, killed his wife, in order, as he declared, to *cook her feet*, and, being disturbed in his horrible meal, he also killed an old man who had accidentally called at his house.

It is certainly remarkable that in the free-air system insane people, under the moral restraint of knowing that they are watched and observed, are less tempted to yield to morbid impulses than otherwise. In a population of a thousand free lunatics in a village, the great number of moral insanity cases is quite sufficient to guarantee the practibility of the system.

Since authors have reported that lunatics experienced, in cases of fits, a sensation of burning, that, when it arrived at the head, caused a momentaneous fever. There is, and I must notice it, much affinity between diastrephia and epilepsy—they pass also into each other, as in the following case:

A young man had suffered eight years of epileptic fits. During two years the fire was within him, but instead he had fits of diastrephia. As soon as he felt the attack he used to cry, *Dear mother, be off, or I must kill you!*

Diastrephia, if cured in its early period, ends generally in dementia or general paralysis. This desease is frequent; but authors describe it under an other denomination. For instance, all the cases died in the manual of Bucknill and Tuke, from p. 178 to 220, under the head of *Emotional Insanity*, are all, except a case of erotomania, pure diastrephic cases.

All the symptoms that we have enumerated are not seen at first sight; they require time and leisure to be detected. It is not to be expected that a physician, suddenly called into a court of justice, would be able to ascertain their presence in a first examination. Days, and sometimes months, may be necessary to investigate doubtful and difficult cases. The necessity of a fixed rule by which medical officers should be under the obligation to specify the moral and physiological signs of insanity in their affidavits is evident. The numberless difficulties arising from summary legal reports, and the dangers resulting from it to individual liberty and property, threaten every one.

In civil lawsuits concerning the suspension of civil rights, the validity of the public transactions of certain persons, the state of mind of persons having made wills and donations, etc., it is often necessary to examine certificates concerning past periods of the life of a person; but if these certificates or documents are defective or incomplete, on account of some general statement giving no details or description, they can be of no use. In criminal prosecutions it is sometimes necessary to know whether insanity existed at a certain time—its nature and its form—sometimes useful documents might be found concerning the parents and ancestors or adoptors of an accused party. One difficult point remains to be elucidated concerning incipient cases of diastrephia, or rather concerning the period of its prodroms. The question is this—When ought vice and immorality to be considered the proximate causes and motive of a reprehensible act? And when ought it to be considered as the result of a diseased brain? We would propose that, if pathological symptoms are not evident, if, at the same time, psychological signs are doubtful, on account of the identity of probable cause and of its results, no medical man should give his evidence in favor of insanity. And the reason is obvious. In a sort of intermediate state we have no right to decide the question.

In the course of this paper we have tried to keep clear of a confusion about the unsoundness of moral sense, widely different from that unsoundness of mind which is the result of a pathological condition of the brain. Physicians have no wish to impose ambiguities by which juries or public opinion should be bewildered. If they differ sometimes in opinion, it depends entirely upon the nature of doubtful cases.

On my arrival at Sing-Sing, I was kindly lent a book on the case of a late broker, Charles Huntington, and through the influence of a most distinguished and deserving physician, Doctor Fisher, residing there, was introduced to the convict in the State Prison.

While reading the case it appeared to me that either the subject had been a lunatic, afflicted with a special deficiency of moral sense, with great instinctive cunning and ability to deceive, or that he was a lunatic under the influence of some kind of diastrephia. After my visit to the prison I came to the conclusion that the prisoner was neither the one or the other, although many facts showed that he had been in the process of becoming really insane.

After a few words were exchanged, the convict himself told me that in his opinion he had never been properly insane, though he had felt something wrong in his head until two years since. Up to that period he would have counterfeited any man's signature. He said his habit of forging had come to such a degree that to get *his own money* out of a bank he would have rather employed a forged paper!

Being, of course, a perfect stranger to all parties who appeared in this trial, the opinion I venture is free from prejudice and only liable to errors of my own.

It appears that in the State of New York it is a jury that decides upon cases when the plea of insanity is brought forward. The law of the State, as explained in the case of Huntington, does not admit moral insanity as an excuse for responsibility.

Partial insanity or monomania would not absolve the party unless it wholly deprived the patient of the power to distinguish right from wrong! We have already objected to such absurd worn-out jurisprudence—more perhaps than 600 years old—in England. But whatever the law may be, by another mistake the jury is omnipotent in scientific difficulties, because by its verdict the jury may either absolve without explanation or condemn without the slighest light on the scientific question. Now I believe that the jury was accidentally right in finding Huntington guilty, although some doubts might have been entertained on his real state of mind.

In this point of the trial I am convinced of the good reasons brought forward by the two medical witnesses to establish their opinion. I do not partake entirely of it, but I acknowledge their sincerity and scientific ground. These physicians have, I may say, to the honor of our profession, shown their profound knowledge and aptitude to discuss difficult points on a medico-psychological specialty; but the whole of their discussion with clever jurists shows how much physical symptoms were wanted to confirm their opinion. It is even curious to remark that their opponent's arguments were based on this very want of symptomatic evidence. However, the whole appearance of the case leads to the admission of a prodromic stage of diastrephia.

The conduct of Huntington from a boy up to the time of his trial bears the characteristic of some morbid diathesis and hereditary taint of insanity. His diseases, when a child, might have had a depressive influence on his conscience; and later, when a man, his temperament and propensities could hardly be checked by mixing with speculators in stock-jobbing. He lived alternately in wealth and poverty, extravagance and want. In prison he was found indifferent to his situation, although accused of a capital offence! His appreciation was certainly defective. Reading the case, one might ask whether he was not stimulating insanity? The prisioner maintained that he never intended injuring people! Still he found in forgeries the means of gathering enormous sums of money, of which he spent a great deal in self-gratification! Huntington said that it was a desire that came over him; that nothing could have prevented him from forging false paper, and that it was only two years since that he felt better, could sleep, and could say that he would not do it again.

The learned counsel for his defense explained the curious circumstance of the carelessness of his client, who had made no arrangements to escape or prevent his arrest.

Then the able advocate put several questions to the physicians. 1st, Whether in their opinion, the defendant was sane or insane when the forgeries were committed? 2d, If insane, what was the nature and character of that insanity?

It was answered that it might be possible that all might take place as the result of almost unparalleled recklessness, but that from personal examination, and also from the testimony heard, it might be said that those actions were the actions of an insane man.

The answer appears unsatisfactory because it admits almost the possibility of recklessness, and does not point out the morbid nature of the acts. The testimonies on Huntington's conduct could not supply the wanted symptoms of an actual state of insanity. The mental state of the prisoner was the first and principal object of demonstration; his actions were naturally the consequence of his mental disposition.

Cross-examined by the advocate of the prosecution, one of the medical gentlemen was asked this question—

Upon what the prisoner said to you, and from what you judged from his appearance, would you pronounce him of unsound mind, from your examination and from his appearance?

The question is direct, and points the vital knot of the case. Besides, this question shuts all issues, by repeating that the source of information must be personal examination of the prisoner.

The answer was—Not by his appearance, but from my examination of him I should.

Now by this answer physical symptoms were almost abandoned as of no value. However, the same physician said further, that the expression of the face of Huntington was not that of a villian but that of an insane man.

This general statement might have been good, if followed by a description which might have convinced the jury and court. Now, the advocate for the prosecution very adroitly asked the doctor to explain, what was the disease of his physical organization which prevented him from resisting the tendency to commit forgery? It was a quibble; but the lawyer knew the weak side of the jury, and was certain of being victorious if he could prevent the physician from giving a satisfactory answer. The physician honestly said that he could not give the pathological anatomy of the case! The advocate insisted upon knowing the relation of a physicial lesion of the brain to a moral perversity, and repeated the question, What urges the patient to forge paper? This insistance was very likely a necessity of the situation, because the learned lawyer would not have purposely laid a sort of trap for his respected and intimate friend, as he called the doctor in his exordium. I believe that such an unqualified question might have misled a less capable and learned physician to invent some new name of special disease having for its characteristic to *forge paper to the amount of half a million of dollars.* The question was ridiculous and improper, because it had nothing specially to do with the trial, as being a question on primary causality in our moral nature, and a *modus operandi* of anatomy.

Another physician, also a learned professor in a medical school, explained with great accuracy why he did not believe in the existence of monomania. He thought that Huntington was insane because his intellectual and moral nature, as well as his propensities, were diseased. The honorable witness stated, that in this case no delusion nor hallucination existed, but only *moral insanity.* He said that Huntington in his moral obliquity, would, perhaps, in the West, have committed criminal acts attended by violence; but that having *satisfied himself* that Huntington was insane he thought it unsafe to say or foretell what an insane person might be inclined to do.

I sincerely regret that this witness did not add objective proofs to his own conviction. Although the verdict was, I believe, a just and a right one, will it not appear injudicious that a jury of laymen should have to appreciate and judge a medical discussion, led by clever but artful gentlemen of the bar? With reference to the knot offered to the jury to untie, I imagine that the honorable jurors may have very well said among themselves that balancing the moral account of the broker, and finding him guilty, his state of mind had little to do with their decision.

I conclude this paper by the following propositions:

1. That the disease called moral insanity is but an affection of the faculty of violation and instinct, attended by physiological symptoms.

2. That the name of moral insanity is defective, because it bears no relation to the cause, symptoms, and results of that disease; and that it misleads the opinion of the bar concerning crimes committed under its influence.

3. That the laws and rules concerning insanity, relating to civil and criminal cases, ought to be put in accordance with the actual state of medical science.

4. That no person ought to be considered as being insane if physical and mental signs cannot be traced and ascertained.

5. That a reform concerning medical certificates is necessary to insure regularity in obtaining from courts or judges orders to detain a person as insane. That no such document be admitted unless containing, 1st, All the moral and physical symptoms of the case; 2d, The diagnosis and prognosis of the disease.

Now, until a reform be made, we should have license to say to the legislators and jurists of this and other countries—Si habetis corpus, nos habemus animam.

Sing-Sing, Oct. 1861.—*Medical Times.*

